# In Situ Raman Investigation of TiO_2_ Nanotube Array-Based Ultraviolet Photodetectors: Effects of Nanotube Length

**DOI:** 10.3390/molecules25081854

**Published:** 2020-04-17

**Authors:** Yanyu Ren, Xiumin Shi, Pengcheng Xia, Shuang Li, Mingyang Lv, Yunxin Wang, Zhu Mao

**Affiliations:** 1 School of Chemistry and Life Science, Advanced Institute of Materials Science, Changchun University of Technology, Changchun 130012, China; ryy17543012961@163.com (Y.R.); x13385955107@163.com (P.X.); lishuang1071418026@163.com (S.L.); lmy2508644298@163.com (M.L.); 2 College of Chemical Engineering, Changchun University of Technology, Changchun 130012, China; shixiumin@ccut.edu.cn; 3 Jilin Provincial Center for Disease Control and Prevention, Changchun 130062, China; xierlian@163.com

**Keywords:** TiO_2_ nanotube arrays, UV photodetector, Raman spectroscopy, Surface-enhanced Raman scattering, SERS

## Abstract

TiO_2_ nanotube arrays (TNAs) with tube lengths of 4, 6, and 7 μm were prepared via two-step anodization. Thereafter, ultraviolet (UV) photodetectors (PDs) with Au/TiO_2_/Au structures were prepared using these TNAs with different tube lengths. The effects of TNA length and device area on the performance of the device were investigated using in situ Raman spectroscopy. The maximum laser/dark current ratio was achieved by using a TNA with a size of 1 × 1 cm^2^ and a length of 7 μm, under a 532 nm laser. In addition, when the device was irradiated with a higher energy laser (325 nm), the UV Raman spectrum was found to be more sensitive than the visible Raman spectrum. At 325 nm, the laser/dark current ratio was nearly 24 times higher than that under a 532 nm laser. Six phonon modes of anatase TNAs were observed, at 144, 199, 395, 514, and 635 cm^−1^, which were assigned to the E_g(1)_, E_g(2)_, B_1g(1)_, A_1g_/B_1g(2)_, and E_g(3)_ modes, respectively. The strong low-frequency band at 144 cm^−1^ was caused by the O-Ti-O bending vibration and is a characteristic band of anatase. The results show that the performance of TNA-based PDs is length-dependent. Surface-enhanced Raman scattering signals of 4-mercaptobenzoic acid (4-MBA) molecules were also observed on the TNA surface. This result indicates that the length-dependent performance may be derived from an increase in the specific surface area of the TNA. In addition, the strong absorption of UV light by the TNAs caused a blueshift of the E_g(1)_ mode.

## 1. Introduction

Highly ordered TiO_2_ nanotube arrays (TNAs) were synthesized for the first time in 2001 [1]. Because of their controllable diameter, uniform morphology, and large specific surface area, they have been widely used in various industries, such as for gas sensors, water light solutions, dye-sensitized solar cells, and electrochromic devices [2,3,4,5,6,7,8]. In addition, because of the large bandgap of TiO_2_ (3.2 eV for anatase and 3.0 eV for the rutile structure) there is no need to filter out visible or infrared light, which is ideal for ultraviolet (UV) detection applications [9,10,11,12,13,14,15]. Many studies have been conducted to improve the performance of TiO_2_-based photodetectors (PDs) [16].

It is essential to understand the surface and interface structures of PDs for the further development and optimization of the performance of UV PDs [17,18,19,20,21,22]. However, the current characterization technologies for PD-related devices are mostly based on electrochemical methods. They can collect the photocurrent–voltage curves and transient photocurrent response relationships of PDs [23,24,25]. Thus, only limited knowledge regarding the surface and interface structures of these devices is available. Therefore, characterization techniques that can provide detailed information about the TiO_2_ structure of a device in situ remain a challenge.

Raman spectroscopy utilizes a scattering spectrum that is based on the Raman scattering effect, discovered by an Indian scientist, Sir C.V. Raman [26]. It is an analytical method that can analyze the scattering spectrum at a frequency different from the incident light in order to obtain molecular vibration and rotation information; it is also used in molecular structure research. Raman spectroscopy is widely used to characterize the crystallinity and crystallographic orientation of metal oxide materials [27]. As with the detection of target molecules, solid materials can be identified by their characteristic phonon patterns. In situ Raman technology has the characteristics of micron-level spatial resolution and non-destructive detection [28,29]. Raman spectroscopy has proven itself to be a powerful characterization technique for obtaining detailed information about the molecular structure of metal oxides [30,31,32]. This is because each molecular state has a unique vibration spectrum related to its structure. Moreover, Raman spectroscopy is suitable for in situ research [33] and can also judge and analyze doping, defects, and small changes in the crystal structure of molecules and crystals. Its advantages include simple sample preparation, quick and easy testing, and nondestructive testing at room temperature.

In this work, a Raman laser was used as both the radiation source of the PD, and the excitation laser of the Raman spectrum, in order to characterize both the photoelectric performance and the Raman spectrum of the PD. The results of this study will help researchers to understand the effect of the TiO_2_ structure on the performance of UV detectors at the phonon level.

## 2. Results and Discussion

**Figure 1:**
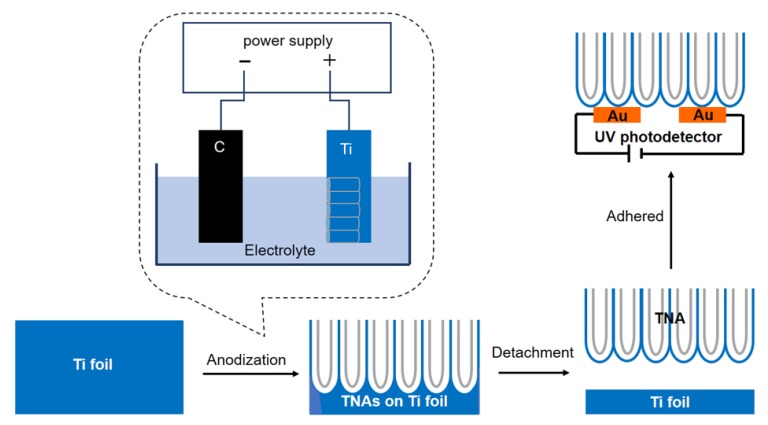
Preparation process of TiO_2_ nanotube array (TNA)-based ultraviolet (UV) photodetectors (PDs).

An illustration of the preparation of the TNAs and PDs is shown in Figure 1. First, the TiO_2_ TNAs were peeled from the Ti foil and then transferred to an Au interdigital electrode. The binder was a TiO_2_ sol. The results show that the PD with a metal–semiconductor–metal (MSM) structure has a good performance because of its Schottky contacts.

**Figure 2:**
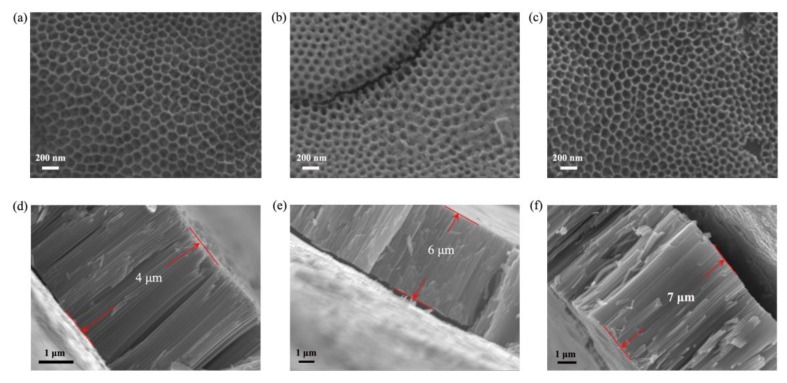
Scanning electron microscopy images of TNAs with different tube lengths: (**a**) top and (**d**) side views with secondary oxidation time of 1 h; (**b**) top and (**e**) side views with secondary oxidation time of 2 h; (**c**) top and (**f**) side views with 3 h oxidation time.

In this work, the length of the tubes was adjusted by changing the time of secondary anodization, and TNAs with secondary oxidation times of 1 h, 2 h, and 3 h were prepared. The scanning electron microscopy (SEM) images shown in Figure 2 reveal that the tube lengths of the TNAs after 1, 2, and 3 h were 4, 6, and 7 μm, respectively, and the average diameter was less than 100 nm. As shown in Figure 2, the surface of the TNAs is flat and clean. As shown in Figure 2, it is obvious that it takes 1 h to grow from 6 μm to 7 μm. As the tube becomes longer, its growth rate will be slower. In addition, it was also found that as the length of the TNA increases, it becomes easier to fracture during the transfer process, and the performance difference caused by this result is also difficult to rule out. Therefore, we have selected tube lengths of 4 μm, 6 μm, and 7 μm for discussion.

**Figure 3:**
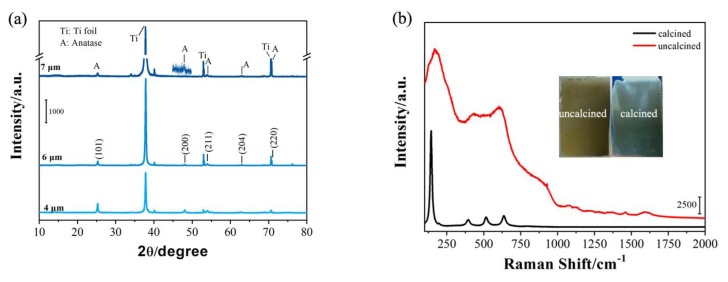
(**a**) X-ray diffraction patterns of TNAs with different tube lengths; (**b**) Raman spectra of uncalcined and calcined TNAs (inset: photographs of uncalcined and calcined TNAs).

Figure 3 shows the X-ray diffraction (XRD) patterns of the TNAs with tube lengths of 4, 6, and 7 μm after calcination at 450 °C. As shown in Figure 3a, the seven peaks at 25.3°, 48.1°, 54.9°, 62.9°, and 70.6° are basically consistent with the corresponding sample structure No. 84-1285 in the JCPDS file and respectively correspond to the (101), (200), (211), (204), and (220) directions, proving that the TNAs are anatases. The results show that as the tube length increases, the intensity of the XRD peaks increases. The Raman spectra of the uncalcined and calcined TNA were characterized. As shown in Figure 3b, the Raman spectrum of the calcined TNA shows distinct anatase characteristic phonon modes. Furthermore, as shown in the photographs in Figure 3b, the TNA before calcination appears yellow, while after calcination, it is white. In addition, many previous publications have stated that a calcined temperature of 450 °C is sufficient to obtain the anatase structure [34,35,36]. The intense E_g(1)_ mode (144 cm^−1^) proves that the main phase of the TNA is anatase.

Four PDs with different areas were prepared using TNAs with tube lengths of 6 μm. The device areas were 2 × 2, 1.5 × 1.26, 2 × 1, and 1 × 1 cm^2^ (the width and spacing of the interdigitated Au were both 100 μm). An excitation wavelength of 325 nm was used for the irradiation light source (the laser power was 6.3 mW) in order to test the laser/dark current response performance of the devices.

**Figure 4:**
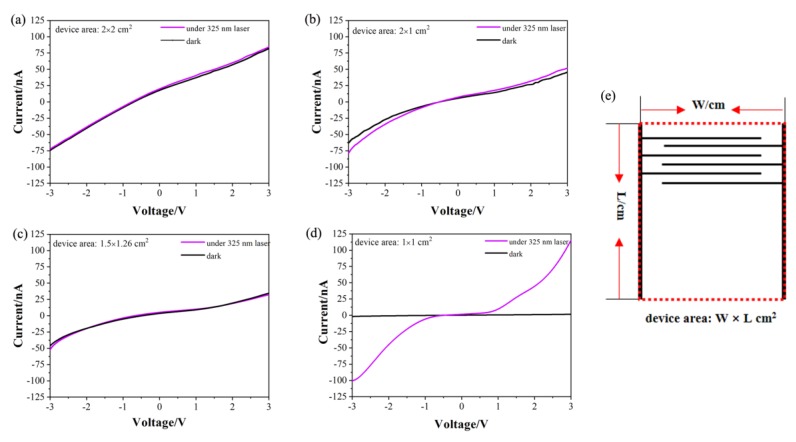
Current-voltage (*I*-*V*) curves of PDs with different device areas: (**a**) 2 × 2 cm^2^; (**b**) 2 × 1 cm^2^; (**c**) 1.5 × 1.26 cm^2^; and (**d**) 1 × 1 cm^2^ under 325 nm laser. (**e**) Schematic view of device area (W × L, red dotted border) on the Au interdigitated electrode.

To clarify the definition of the device area, a schematic view of the device area on the Au interdigitated electrode is shown in Figure 4e. On the premise of keeping the width and spacing of the interdigitated electrode at 100 μm, we changed the device area by changing the length and/or the number of the interdigitated Au. As shown in Figure 4e, the red dotted border represents the typical device area. In the preparation of large-area devices, the TNA film may inevitably break during the transfer process, resulting in reduced conductivity. This is the main reason for the serious degradation of device performance. Therefore, in this work, the main purpose of exploring the device area effect is to indirectly screen out the TNA film area that, in some cases, provides a better performance.

As shown in Figure 4, the laser/dark current ratios of the PDs were calculated under a bias of 3 V. The results show that the laser/dark current ratios of the PDs with device areas of 2 × 2, 1.5 × 1.26, 1 × 2, and 1 × 1 cm^2^ were 1.03, 1.07, 1.13, and 79.70, respectively. As shown in Figure 4d, the dark current of the PD with an area of 1 × 1 cm^2^ was 1.447 nA, and its photocurrent was 115.30 nA. The results show that the laser/dark current ratio of the device with the area of 1 × 1 cm^2^ was the largest, indicating that this device has the highest light responsivity at 325 nm. Therefore, the PD with an area of 1 × 1 cm^2^ was used for subsequent experiments.

To explore the impact of laser energy on the performance of a PD, 532 nm and 325 nm lasers were used to simultaneously detect the laser/dark current ratio and Raman spectra of the PD with an area of 1 × 1 cm^2^. The 532 nm laser was used with a 50 × objective lens (NA 0.5), and the 325 nm laser was used with a 15 × objective lens (NA 0.32). The spot sizes were approximately 1.30 μm for the 532 nm laser, and 1.24 μm for the 325 nm laser (spot size = 1.22 λ/NA).

**Figure 5:**
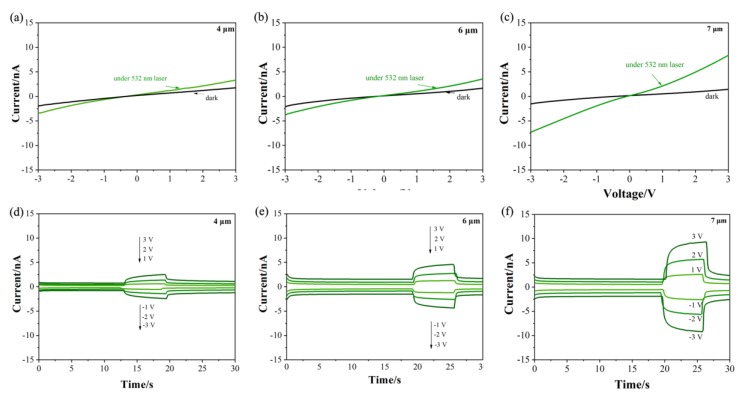
Performance of TNA-based PDs with different tube lengths in the dark and under a 532 nm laser: (**a**) 4 μm; (**b**) 6 μm; (**c**) 7 μm. Fast response measurements with different tube lengths: (**d**) 4 μm; (**e**) 6 μm; (**f**) 7 μm. (laser power: 5.3 mW).

Firstly, a 532 nm laser was used to study the effect of length on the PD performance. Figure 5 shows the I–V curves and the fast-response measurements of TNA-based PDs with different tube lengths. Here, when the bias was 3 V, the laser/dark current ratios of the PDs with tube lengths of 4, 6, and 7 μm were 1.89, 2.14, and 5.89, respectively. The results show that as the length of the TNA tube increases, the laser/dark current ratio of the device increases accordingly. This result indicates that as the length of the tube increases, the transfer of photogenerated charge increases—that is, as the length of the tube increases, the light response of the device also increases.

**Figure 6:**
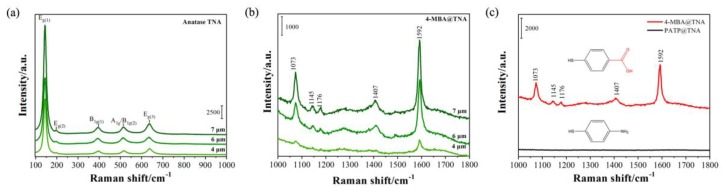
(**a**) In situ Raman spectra of TNA with different tube lengths; (**b**) surface-enhanced Raman scattering spectra (SERS) of 4-mercaptobenzoic acid (4-MBA)-modified TNA with different lengths; (**c**) Raman spectra of 4-MBA- and 4-aminothiophenol (PATP)-modified TNAs. The wavelength of excitation is 532 nm.

Secondly, the in situ Raman spectra of TNA-based PDs with different tube lengths were collected using a 532 nm laser. The tetragonal anatase phase of a TNA has six Raman-active phonons in the vibration spectrum: 3E_g_ + 2B_1g_ + A_lg_. As shown in Figure 6, all six modes were observed at 144, 199, 395, 514, and 635 cm^−1^, which were assigned to the E_g(1)_, E_g(2)_, B_1g(1)_, A_1g_/B_1g(2)_, and E_g(3)_ modes, respectively. The strong low-frequency band at 144 cm^−1^ was caused by the O-Ti-O bending vibration and is a characteristic band of anatase. As shown in Figure 6a, as the tube length increased, the intensity of the E_g(1)_ mode increased, indicating that the E_g(1)_ mode of the TNA has a tube length-dependent effect.

Moreover, as shown in Figure 6c, it is clear that the Raman signal of 4-mercaptobenzoic acid (4-MBA) molecules was enhanced on the surface of the TNA, but the Raman signal of 4-aminothiophenol (PATP) molecules was not enhanced. The results indicate that there were abundant hydroxyl groups on the surface of the TNA, which can strongly interact with the carboxyl groups of the 4-MBA molecules, thereby promoting the charge transfer resonance between the TNA and 4-MBA. In addition, the surface-enhanced Raman scattering (SERS) [37] activity of the TNA has a length-dependent effect. As shown in Figure 6b, as the length of the TNA increased, the SERS intensity of 4-MBA increased; more specifically, when the tube length was 7 μm the SERS signal was at its highest. The surface area is proportional to the volume of the tubes. However, it has been unanimously agreed in previous publications that the oxidation voltage is the main factor affecting the diameter of the TNA [38,39,40]. Moreover, the oxidation time is the main factor that affects the length of the TNA. In this work, to highlight the influence of the TNA length, the oxidation voltage is always constant (i.e., 60 V). In Figure 2, it is clear that there is almost no difference in the diameter of the TNAs. As the oxidation time increases, the length of the prepared TNA also increases, resulting in an increase in the specific surface area; moreover, the number of molecules adsorbed on the TNA surface increases, which ultimately leads to an increase in SERS activity. The high specific surface area of the 7 μm TNA also influences the light response performance of the TNA-based PD.

The intense bands at approximately 1592 and 1073 cm^−1^ can be assigned to totally symmetric C–C stretching and in-plane ring breathing, respectively [41,42]. From the SERS spectra of the 4-MBA-adsorbed TNA, other weak bands were also observed, corresponding to the C–H deformation modes at 1145 and 1176 cm^−1^, respectively [41,43]. A more detailed assignment of the Raman bands of 4-MBA is shown in Table 1. It is clear from Figure 6b that when the length of the TNA was 7 μm a significantly enhanced SERS signal was obtained. In this case, the enhanced SERS signal likely originated from the charge transfer (CT) effect.

**Figure 7:**
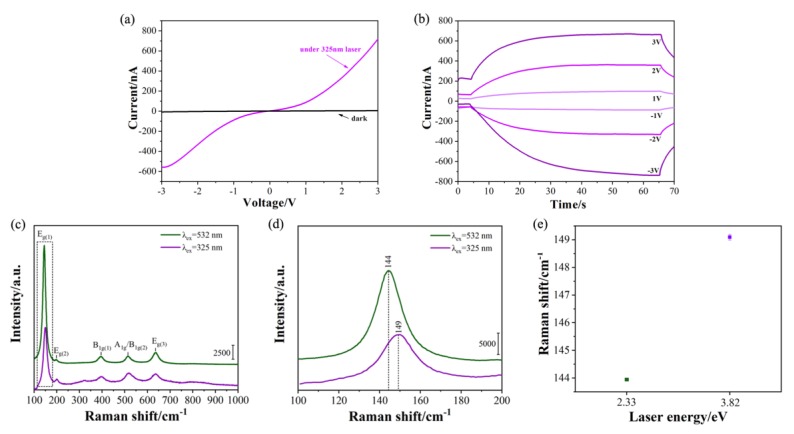
(**a**) *I*-*V* curve and (**b**) fast-response measurements of a TNA-based PD with a tube length of 7 μm under 325 nm excitation; (**c**) in situ Raman spectra of TNA-based PD collected at excitation wavelengths of 532 nm and 325 nm; (**d**) enlarged Raman spectrum in the 110–200 cm^−1^ region; (**e**) The reproducibility of Raman measurements.

Finally, the performance of a TNA-based PD with a tube length of 7 μm was tested using a 325 nm laser. As shown in Figure 7, the results show that when the applied bias was 3 V, the laser/dark current ratio of the device was 139.1, which is almost 24 times larger than that under a 532 nm laser.

With an excitation wavelength of 325 nm, the phonon modes of E_g(1)_, E_g(2)_, B_1g(1)_, A_1g_/B_1g(2)_, and E_g(3)_ were observed. It is worth mentioning that the frequency of the E_g(1)_ mode shifted to 149 cm^−1^ compared with that for 532 nm excitation. This result suggests that the value of the laser energy (3.82 eV) is greater than the bandgap of the TNA, which causes photons to effectively interact with the phonons of TNA. This increase in frequency was derived from resonant Raman scattering. This work demonstrates the ability of in situ Raman technology to reveal the internal relationships between device interface structure and performance.

## 3. Materials and Methods

### 3.1. Chemicals

Ammonium fluoride, hydrogen fluoride, and n-butyl titanate were purchased from Macleans Corporation. Ethylene glycol, hydrogen peroxide, and isopropanol was purchased from Tianjin Fuyu Fine Chemical Co., Ltd. (Tianjin, China). Ethanol, hydrochloric acid, acetone, and methanol were purchased from Beijing Chemical Industry Group Co., Ltd. (Beijing, China). The abovementioned reagents were used without further purification. The water used in the experiment was ultrapure water. 4-MBA and PATP were purchased from Sigma-Aldrich Co., Ltd. (St. Louis, MO, USA) and used without further purification.

### 3.2. Instruments

SEM characterization was performed using a Shimadzu SSX-550 scanning electron microscope: the acceleration voltage was 3.0 kV. XRD was performed using a Japanese Rigaku Smartlab X-ray diffractometer with Cu-Kα rays (λ = 1.5418 Å) at 45 kV and 200 mA. The Raman spectra were measured using a Horiba JY LabRAM HR Evolution confocal micro-Raman spectroscopy equipped with a multichannel air-cooled charge-coupled device detector. The 532 nm and 325 nm lasers were employed as excitation laser sources. The Raman spectra for an excitation of 532 nm were measured using a 50× objective lens, 5.3 mW laser power at the sample, and 1800 gr/mm grating. The Raman spectra for the 325 nm excitation were measured using a 15× objective lens, 6.3 mW laser power at the sample, and 2400 gr/mm grating.

### 3.3. Preparation of TiO_2_ Nanotube Arrays

First, the Ti foil was cut to an area of 4 × 5 cm^2^ and ultrasonically cleaned in acetone, isopropanol, and methanol solutions for 10 min to remove impurities on the surface. Thereafter, the Ti foil was dried using an N_2_ flow. TNA was prepared via anodic oxidation using an ethylene glycol solution of NH_4_F and deionized water (0.3% NH_4_F + 2% H_2_O + 500 mL ethylene glycol) as the electrolyte, with Ti foil as the anode, and a graphite sheet as the cathode.

Anodization was performed twice, and the anodizing process is depicted in Figure 1. The first oxidation time was 3 h with a voltage of 60 V. The first TNA film obtained was peeled from the Ti foil and rinsed with a large amount of deionized water in order to obtain a regular and ordered Ti substrate, after which it was dried with N_2_ flow. The second oxidation process was performed for either 1 h, 2 h, or 3 h at a voltage of 60 V. The TNA samples of different lengths were then calcined at 450 °C for 2 h to obtain the anatase phase.

### 3.4. Preparation of TiO_2_ Sol

A set amount of 5.00 mL of n-butyl titanate and 9 mL of ethanol were mixed and stirred well to form component A. Then, 18 mL of ethanol, 1 mL of HCl, and 1 mL of H_2_O were mixed and stirred well to obtain component B. Then, component A was added to component B and stirred for 4 h to obtain a yellow TiO_2_ sol.

### 3.5. Preparation of the TiO_2_ Nanotube Array-Based Ultraviolet Photodetector

To prepare an MSM-structured PD, the calcined TNA was stripped. Then, the TNA was oxidized at 60 V for 20 min. Thereafter, the TNA was cleaned and immersed in 40% H_2_O_2_ for 15 min to separate the TNA film from the Ti foil. The TiO_2_ sol was then dropped on the Au interdigital electrode with Al_2_O_3_ as a substrate, and the peeled TNA film was transferred to the Au interdigital electrode. Following this, the film was calcined at 450 °C for 2 h. After annealing, the TNA film was located perpendicular to the Au interdigital electrode as an MSM structure, as shown in Figure 1.

## 4. Conclusions

In this work, TNA-based PDs were prepared with TNA lengths of 4, 6, and 7 μm. Firstly, the effect of the device area (2 × 2, 1.5 × 1.26, 2 × 1, and 1 × 1 cm^2^) on the performance of the laser response was investigated. The results show that the device with an area of 1 × 1 cm^2^ exhibited the best performance. Secondly, we analyzed the effect of tube length on the performance of the laser response. The results show that the device with the longest (7 μm) tube had the best laser response performance, with a laser/dark current ratio of 5.89. At 325 nm, the laser/dark current ratio was 139.1. In addition, as the tube length increased, the strength of phonon mode E_g(1)_ increased, indicating that the strength of the E_g(1)_ mode is length-dependent. Under irradiation of a 325 nm laser, the E_g(1)_ mode shifted to a higher frequency by 5 cm^−1^ compared with that under the 532 nm laser. The SERS signals of 4-MBA molecules were also observed on the TNA surface. This result indicates that the length-dependent performance may be derived from the increase in the specific surface area of the TNA. Furthermore, in situ Raman spectroscopy successfully revealed the dependence of the phonon mode of TiO_2_ on the laser energy and TNA length of a TNA-based PD. The results of this study may help researchers understand the effects of the TiO_2_ structure on the performance of UV detectors from a phonon perspective.

## Figures and Tables

**Table 1:** Raman bands and assignments of 4-MBA-modified TNA.

Raman Band	Assignment
144 cm^−1^	E_g(1)_ (Anatase TiO_2_)
199 cm^−1^	E_g(2)_ (Anatase TiO_2_)
395 cm^−1^	B_1g(1)_ (Anatase TiO_2_)
514 cm^−1^	A_1__g_/B_1g(2)_ (Anatase TiO_2_)
635 cm^−1^	E_g(3)_ (Anatase TiO_2_)
1073 cm^−1^	in-plane ring breathing + C−S stretching (4-MBA)
1148 cm^−1^	C−H deformation (4-MBA)
1176 cm^−1^	C−H deformation (4-MBA)
1407 cm^−1^	COO^-^ stretching (4-MBA)
1592 cm^−1^	totally symmetric C−C stretching (4-MBA)
